# Affino-Proteomic Analysis of Bumped Kinase Inhibitor BKI-1708 in *Toxoplasma gondii* and Human Fibroblast Host Cells

**DOI:** 10.3390/microorganisms14081608

**Published:** 2026-07-23

**Authors:** Maria Cristina Ferreira de Sousa, Joachim Müller, Manfred Heller, Anne-Christine Uldry, Sophie Braga-Lagache, Kayode K. Ojo, Wesley C. Van Voorhis, Andrew Hemphill

**Affiliations:** 1 Institute of Parasitology, Vetsuisse Faculty, University of Bern, 3012 Bern, Switzerland; joachim.mueller@unibe.ch; 2 Graduate School for Cellular and Biomedical Sciences (GCB), University of Bern, 3012 Bern, Switzerland; 3 Proteomics and Mass Spectrometry Core Facility, Department for BioMedical Research (DBMR), University of Bern, 3012 Bern, Switzerland; manfred.heller@unibe.ch (M.H.); anne-christine.uldry@unibe.ch (A.-C.U.); sophie.braga@unibe.ch (S.B.-L.); 4 Department of Medicine, Center for Emerging and Re-Emerging Infectious Diseases, University of Washington, Seattle, WA 98109-4766, USA; ojo67kk@uw.edu (K.K.O.); wesley@uw.edu (W.C.V.V.)

**Keywords:** *Toxoplasma gondii*, drug treatment, bumped kinase inhibitors, affinity chromatography, proteomics, drug targets

## Abstract

Bumped kinase inhibitor 1708 (BKI-1708), previously demonstrated to target apicomplexan kinases and CDPK1 and MAPKL1, exhibits remarkable activity against *Toxoplasma gondii* infection both in vitro and in vivo. Notably, BKI-1708 does not affect the viability of mammalian cells. Upon exposure to BKI-1708, *T. gondii* tachyzoites form large multinucleated complexes named baryzoites and remain trapped within host cells. In this study, proteins binding to BKI-1708 were identified in soluble extracts of *T. gondii* ME49 tachyzoites and human foreskin fibroblasts (HFF) using differential affinity chromatography coupled to mass spectrometry (DAC-MS). Beyond kinases, secondary interactions in *T. gondii* involved the binding of proteins associated with cell division, cytoskeleton, vesicular trafficking, secretory organelles, and transcriptional and translational regulators. In non-infected HFFs, BKI-1708 interactors included cytoskeletal regulators along with multiple RNA/DNA-binding proteins. Upon infection, this profile shifted, with cytoskeletal components no longer detected, while nucleic acid-binding proteins remained present, consistent with infection-induced chromatin and transcriptional remodeling. These results suggest that multi-target interference could contribute to the impaired cytokinesis and the formation of multinucleated baryzoites, aligning with the concept that antiprotozoal drugs exert efficacy through coordinated perturbation of multiple cellular processes rather than a single dominant target.

## 1. Introduction

*Toxoplasma gondii* is an obligate intracellular apicomplexan parasite of significant medical and veterinary importance, being the causative agent of the globally prevalent zoonosis toxoplasmosis [1]. Its life cycle is facultatively heteroxenous, with sexual reproduction confined to felids, and gamogony and oocyst formation taking place in the intestinal epithelium [2]. Numerous intermediate hosts, particularly man and small ruminants, acquire infection by consuming sporulated oocysts from contaminated food or water, or via undercooked meat harboring tissue cysts [1,3,4]. After invasion of enterocytes in the small intestine, parasites differentiate into rapidly replicating tachyzoites that disseminate throughout the host, infecting various organs. In immunocompetent individuals, tachyzoites differentiate into slow-growing bradyzoites, forming dormant tissue cysts in muscle and immunologically privileged sites such as the central nervous system and the eye [5] and establish chronic infection [1,6]. Clinically severe toxoplasmosis primarily affects immunocompromised hosts due to uncontrolled tachyzoite replication or reactivation of latent infection. Primary infection during pregnancy can lead to congenital toxoplasmosis, often resulting in abortion, stillbirth, or severe developmental abnormalities in the fetus [7,8]. Current treatment options are limited by toxicity, poor tolerability, and incomplete efficacy against chronic tissue cysts [9]. The absence of safe, well-tolerated, and cyst-targeting drugs underscores the critical need for the development of novel anti-*Toxoplasma* compounds with enhanced efficacy, reduced side effects, and activity against both acute and chronic stages of the parasite.

Bumped kinase inhibitors (BKIs) represent a promising class of compounds with antiprotozoal activity and in vivo safety [10,11,12]. BKIs were designed based on a pyrazolopyrimidine (PP) or a 5-aminopyrazole-4-carboxamide (AC) scaffold, representing two principal chemical backbones within this compound class [10,11]. Compounds have been optimized to selectively target calcium-dependent protein kinase 1 (CDPK1), a kinase conserved among apicomplexans but absent in mammalian hosts [11,13]. Exploiting the naturally occurring small gatekeeper residue in the ATP-binding of apicomplexan CDPK1 enables selective inhibition, since mammalian kinases have a bulkier gatekeeper residue preventing BKI binding [11]. CDPK1 is required for critical cellular processes such as microneme secretion, gliding motility, host cell invasion, and egress [14,15,16]. Recent studies have revealed that BKIs also inhibit *T. gondii* mitogen-activated protein kinase-like 1 (TgMAPKL1), a kinase that regulates centrosome duplication and is essential for proper endodyogeny [17]. Mutations in TgMAPKL1 disrupt cell division, compromising parasite replication [18]. Moreover, it has been reported that in vitro treatment of several apicomplexans cultured in HFF or in monkey kidney epithelial cell line (MARC) with various BKIs not only impaired host cell invasion and egress but also induced the transformation of intracellular tachyzoites into schizont-like multinucleated complexes (MNCs) [12,19]. These MNCs are characterized by: (i) continuous nuclear division without complete cytokinesis; (ii) accumulation of newly formed intracellular zoites not fully individualized that lack the outer plasma membrane; and (iii) simultaneous display of bradyzoite and tachyzoite features. BKI-induced MNCs are unable to undergo egress from the host cell and can persist in vitro for extended periods. Parasites reconverted to tachyzoites upon drug withdrawal. Designated “baryzoites” (from the Greek βαρύς, meaning massive or inert), this drug-induced stage appears to facilitate parasite survival during prolonged exposure to elevated BKI concentrations [20,21].

BKI-1708 is a bumped kinase inhibitor with a 5-aminopyrazole-4-carboxamide (AC) scaffold [10]. It is effective and safe in vitro and in vivo in pregnant mice infected with *T. gondii* and *Neospora caninum* [22], and is also highly effective against *Cryptosporidium parvum* infection in neonatal mouse and calf models [23], although in *Cryptosporidium*, no MNCs were formed. Recently, a comparative study on baryzoites induced by BKI-1708 in the closely related cyst-forming apicomplexans *T. gondii*, *N. caninum*, and *B. besnoiti* reported common, but also distinguishing features in the three species [24]. TEM analysis showed that all three formed viable intracellular MNCs, but an electron-dense cyst wall-like structure was reported only in *T. gondii* baryzoites, and species-specific differences in antigen expression were observed by immunofluorescence. Comparative proteomic analysis revealed a downregulation of ribosomal proteins, proteins associated with secretory organelles, as well as of transcription and translation factors in baryzoites across all species. Bradyzoite-specific markers were upregulated only in *T. gondii* baryzoites [24]. While BKI-1708 has been shown to target CDPK1 and MAPKL1, its precise molecular mechanism of action remains to be completely resolved, and potential effects on part of the host cells have not been thoroughly evaluated.

To define putative molecular targets in both parasite and host contexts, drug affinity chromatography coupled to mass spectrometry (DAC-MS) was performed. DAC-MS is a chemo-proteomic approach in which the compound of interest is immobilized on a solid matrix and used to enrich interacting proteins from complex lysates, followed by mass spectrometric identification. This strategy enables unbiased capture of both direct binders and associated protein complexes [25]. BKI-1708 has structural similarities to the first-generation antiprotozoal quinine, a compound historically linked to significant off-target effects. A side-by-side structural representation emphasizes the potential for broader target engagement beyond canonical kinase inhibition [26,27]. An earlier DAC-MS study on cell-free extracts of *N. caninum* and *Danio rerio* employing BKI-1748, a compound closely related to BKI-1708, and quinine, showed that a majority of BKI-1748 binding proteins were involved in nucleic acid binding and modification, in particular, RNA splicing and key steps of intermediate metabolism [27]. Moreover, BKI-1748 DAC-MS of extracts of *C. parvum* and of human colon tumor (HCT) host cells revealed the presence of RNA-binding and ribosomal proteins involved in translation and RNA processing in parasite and in host cell eluates from both BKI-1748 and quinine columns, but failed to identify any *C. parvum* proteins binding specifically to BKI-1748 and not to quinine [28]. These results suggested that the molecular interactions of BKI-1748 are not limited to specific targets in apicomplexans such as CDPK1 and MAPKL1, but also to host cell proteins, which are involved in common, essential pathways. This aligns with the evolving paradigm shift from the classical “one drug–one target” model toward a systems-level view, in which a compound engages a network of proteins rather than a single defined enzyme [25]. Evidence from various drug-adapted *T. gondii* strains further supports this concept, demonstrating that resistance to antiprotozoal drugs frequently involves coordinated alterations in multiple proteins and compensatory pathways rather than a single target mutation [29,30]. Here we present the identification of molecular targets of compound BKI-1708 identified by DAC-MS in both *T. gondii* ME49 tachyzoites and *T. gondii*-infected and non-infected human foreskin fibroblast host cells (HFF).

## 2. Materials and Methods

### 2.1. Parasites, Culture Medium, Biochemicals, and Compounds

If not stated otherwise, all tissue culture media were purchased from Gibco-BRL (Zürich, Switzerland) and biochemicals from Sigma (St. Louis, MO, USA). BKI-1708 was synthesized in the Department of Biochemistry of the University of Washington, USA [11] and scaled up by WuXi Apptec Inc., Wuhan, China, to >98% purity by LC/MS-MS and NMR, being provided as powder stored at room temperature. Human foreskin fibroblasts (HFF; ATCC, PCS-201-010TM) were maintained in Dulbecco’s modified Eagle medium (DMEM) supplemented with 10% heat-inactivated and sterile-filtered fetal calf serum (FCS), 50 U of penicillin/mL, and 50 μg streptomycin/mL. *T. gondii* ME49 tachyzoites were cultured as previously described [19]. The chemical structures of BKI-1708 and quinine were designed using ACD/ChemSketch (Freeware) version 2025.2.1; https://www.acdlabs.com/resources/free-chemistry-software-apps/chemsketch-freeware/ (accessed on 15 July 2026).

### 2.2. SEM and TEM of T. gondii Baryzoites Formed upon Exposure to BKI-1708

Drug treatments and preparation for TEM and SEM were done as described by [24]. HFF were grown to confluence in T25 flasks in culture medium at 37 °C/5% CO_2_ and were infected with 1 × 10^6^ *T. gondii* ME49 tachyzoites. At 4 h post-infection, the medium was supplemented with 2.5 µM BKI, or not. Medium was removed after 48 h for untreated cultures, or after 6 days of continuous BKI-1708 treatment. Subsequently, cultures were fixed for TEM and SEM, and processed and embedded in Epon 812 epoxy resin (Sigma, St. Louis, MO, USA) [24]. Sections of 80 nm thickness were cut on an ultramicrotome (Reichert and Jung, Vienna, Austria) and were transferred onto formvar–carbon-coated 200 mesh nickel grids (Plano GmbH, Marburg, Germany). They were stained with Uranyless^®^ and lead citrate (Electron Microscopy Sciences, Hatfield, PA, USA), and specimens were inspected on a FEI Morgagni TEM equipped with a Morada digital camera system (12 Megapixel) operating at 80 kV.

### 2.3. Protein Extraction and Differential Affinity Chromatography (DAC)

For protein extraction, frozen pellets of *T. gondii* ME49 tachyzoites, *T. gondii*-infected HFF, and non-infected HFF were resuspended in ice-cold extraction buffer, i.e., PBS (phosphate-buffered saline) containing 1% Triton X-100 and 1% of Halt proteinase inhibitor cocktail (ThermoFisher, Waltham, MA, USA). Suspensions were vortexed thoroughly and centrifuged (13,000 rpm, 10 min, 4 °C). Extraction of pellets was repeated twice. Four mL of extraction buffer was used in total. Supernatants were combined (resulting in approximately 3 mg of total protein) and subjected to affinity chromatography. To produce the sepharose matrices conjugated to BKI-1708 or quinine, 0.5 g of lyophilized epoxy-sepharose with a C12 spacer was suspended in 15 mL H_2_O and centrifuged at 300× *g* for 5 min. Washes in water were repeated twice, followed by a wash with coupling buffer (0.1 M NaHCO_3_, pH 9.5). After the last wash, 20 mg of each compound dissolved in 2.5 mL DMSO (dimethylsulfoxide) was added, and coupling buffer was added to a maximum volume of 5 mL. Mock column medium was generated by incubating 0.5 g of epoxy-sepharose with DMSO. The mixture was incubated for 3 days at 37 °C under slow but continuous shaking to allow coupling of the epoxy group to the compounds. The resulting column medium (approximately 2 mL) was washed with coupling buffer (15 mL), followed by a wash with ethanolamine (1 M, pH 9.5) and by an incubation in 10 mL of ethanolamine for 4 h at 20 °C in the dark to block residual reactive groups. Then, the column medium was transferred to a chromatography column (Novagen, Merck, Darmstadt, Germany) and extensively washed with PBS-DMSO (1:1) and PBS to remove unbound compounds. The columns were stored in PBS containing 0.02% NaN_3_ at 4 °C. Prior to affinity chromatography, mock columns were combined with columns in tandem (mock first, then compound) and washed with 50 mL PBS equilibrated at 20 °C.

For DAC, mock and compound columns were mounted in parallel. In order to simultaneously obtain quinine and BKI-1708 binding proteins, 2 mL aliquots of crude extracts were loaded in parallel on the mock columns on top of the quinine and BKI-1708 columns and allowed to percolate through the tandem by gravity flow. Subsequently, the tandem was washed with 25 mL PBS equilibrated at 20 °C. Then, the columns were separated, and bound proteins were eluted with 50 mM acetic acid (5 mL per column) by gravity flow. The eluates were lyophilized, and the lyophilizates were stored at −80 °C.

### 2.4. Proteomic Analysis of the Eluted Proteins by Mass Spectrometry

The lyophilized residues were resuspended in 10 μL of 8 M urea and 0.1 M Tris-HCl^−^ (pH = 8), and sonicated in a water bath for 5 min. Proteins were reduced and alkylated with 10 mM dithiothreitol for 30 min at 37 °C and 50 mM iodoacetamide for 30 min at room temperature. Iodoacetamide was quenched by the addition of 5 μL 0.1 M dithiothreitol in 0.1 M Tris-HCl^−^ (pH = 8), and the urea concentration was diluted to 4 M by the addition of 2 mM Calcium dichloride in 20 mM Tris buffer. Proteins were double digested for 2 h at 37° with 1 μL of 0.1 μg/μL LysC sequencing grade protease (Promega, Madison, WI, USA), followed by further dilution of urea to 1.6 M and 1 μL of 0.1 μg/μL trypsin sequencing grade (Promega) overnight at room temperature. Digestion stopped with 1% (*v*/*v*) trifluoroacetic acid end concentration. The digest was spun for 1 min at 16,000 g, after an incubation for 15 min at room temperature, and the cleared supernatant was transferred to a HPLC vial for subsequent nano-liquid reversed phase chromatography coupled to tandem mass spectrometry on a system consisting of a nanoElute 2 ultra-performance liquid chromatograph coupled to a trapped ion mobility spectrometry-time of flight mass spectrometer (timsTOF HT; Bruker Daltonics, Bremen, Germany), through a CaptiveSpray source (Bruker Daltonics, Bremen, Germany) with an end-plate offset of 500 V, a drying temperature of 200 °C, and with the capillary voltage fixed at 1.6 kV. A volume of 2 µL protein digest was loaded onto a pre-column (C18 PepMap 100, 5 µm, 100 A, 300 µm i.d. × 5 mm length, ThermoFisher Scientific, Waltham, MA, USA) at a pressure of 80 bars with 0.05% TFA in water/acetonitrile 98:2. After loading, peptides were eluted in back flush mode onto a PepSep column (150 μm × 15 cm, PepSep, Odense, Denmark) applying a 30 min active peptide separation gradient at a flow rate of 500 nL/min. Data were acquired in a data-dependent acquisition (DDA) method employing eight parallel accumulation-serial fragmentation ramps in the ion mobility range of 0.75 to 1.35 1/k0, *m*/*z* range between 100 and 1700 u at a cycle time of 0.95 s at a 300 Hz acquisition rate.

The mass spectrometry data were searched and quantified with FragPipe, version 22.0 [31] against *T. gondii* ME49 annotated protein sequences concatenated with the Swissprot human sequences release from 2025_01 and some common contaminants, including trypsin/LysC, keratins, bovine serum albumin, etc., and their corresponding reversed sequences. Search parameters included Acetyl (Protein N-term) and Oxidation (M) as variable modifications (3 max variable modifications), Carbamidomethyl (C) as fixed modification, and Trypsin/P (max 3 missed cleavages) as digestion enzyme. MSBooster called the Prosit_2019_irt and Prosit_2023_intensity_timsTOF retention time and spectra prediction models, then peptide spectrum matches, peptides, and proteins were filtered to a 1% false discovery rate using percolator and protein prophet. Protein groups with fewer than two peptides per group were excluded from further data consideration. The leading protein selected by protein prophet was chosen per protein group to calculate an IBAQ (intensity-based absolute quantification) value [32]. From IBAQ values, relative abundance (rAbu) was calculated so that the sum of all rAbu was 1,000,000 for each sample. The putative functions of identified proteins were identified based on information given by Uniprot (www.uniprot.org), Toxo DB (https://toxodb.org), and related databases (accessed on 1 September 2025).

## 3. Results

### 3.1. Exposure of T. gondii Tachyzoites to BKI-1708 Initiates Baryzoite Formation

**Figure 1:**
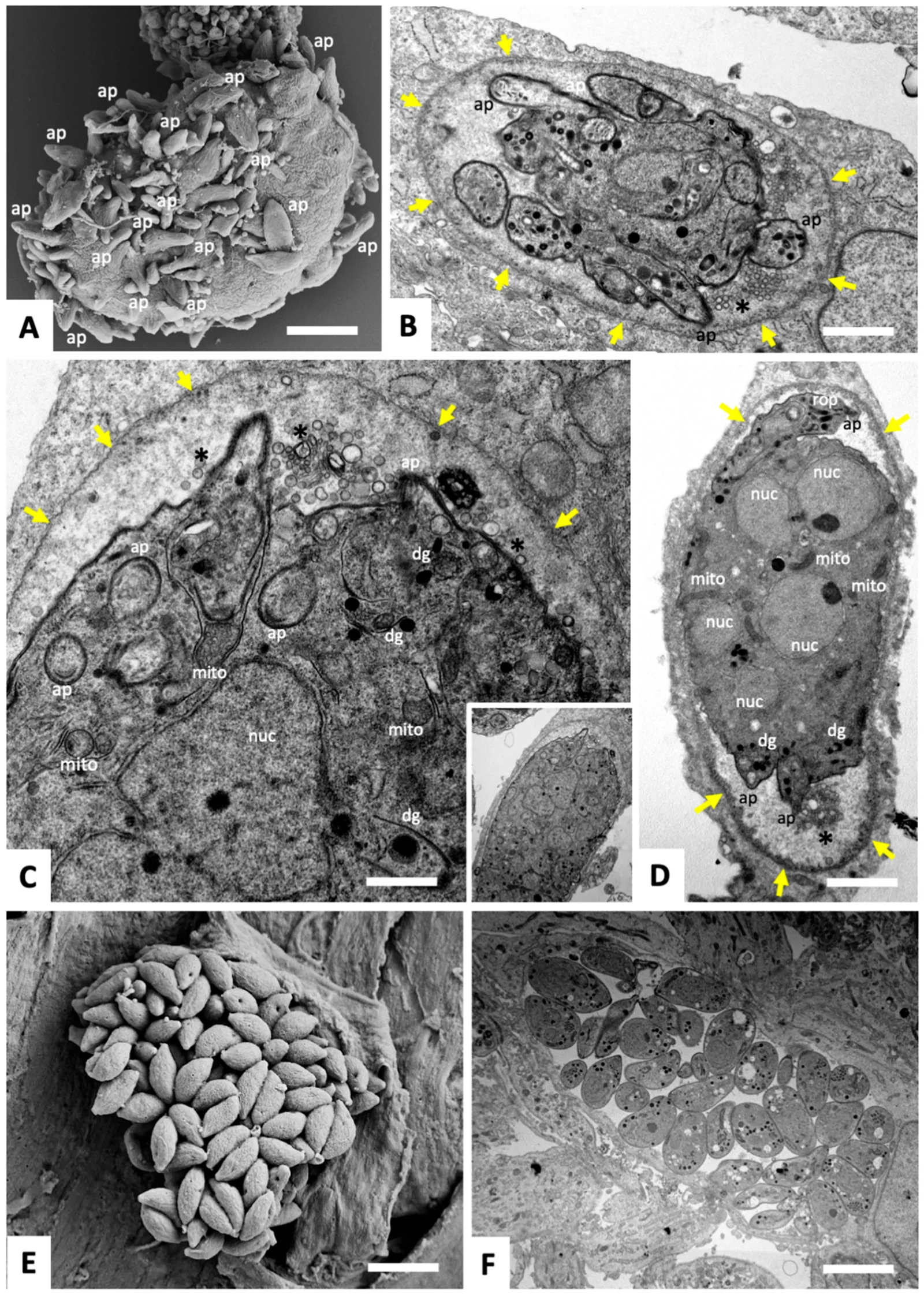
Baryzoite formation in *Toxoplasma gondii* induced by BKI-1708. (**A**–**D**) show infected HFF that were treated with 2.5 µM BKI-1708 starting at 4 h post-infection and were maintained for 6 days prior to processing for SEM (**A**) and TEM (**B**–**D**), the insert in (**C**) provides a lower magnification view of the MNC. (**E**,**F**) show non-treated controls with tachyzoites situated within a parasitophorous vacuole, fixed and processed for SEM and TEM, respectively, after 48 h of culture. Note the multinucleated complexes in BKI-1708-treated cultures, while non-treated cultures contain individual tachyzoites; mito = mitochondrion; nuc = nucleus, ap = apical parts of newly formed zoites; dg = dense granule; rop = rhoptries; arrows point towards electron-dense cyst wall-like structure forming in the parasitophorous vacuole periphery; * indicates secreted material in the vacuole matrix. Bars in (**A**) = 5 µm, (**B**) = 1.9 µm, (**C**) = 0.9 µm, (**D**) = 2.6 µm, (**E**) = 5 µm, (**F**) = 4.2 µm.

HFF were infected with *T. gondii* tachyzoites, and at 4 h post-infection, cultures were treated with 2.5 µM BKI-1708 for a period of 6 days. The resulting baryzoites that were formed upon exposure to BKI-1708 are shown in Figure 1A–D. They form a bulky mass containing numerous nuclei, indicating that DNA replication and nuclear division were maintained. They had different shapes and sizes, often with protruding apical complexes of newly formed zoites pointing outwards. Baryzoites were always surrounded by a triple membrane as found in non-treated tachyzoites, but the internal zoites were clustered together, being inhibited in the completion of cytokinesis and unable to dissociate from each other. The parasitophorous vacuole containing *T. gondii* baryzoites was surrounded by a peripheral electron-dense layer reminiscent of tissue cyst wall formation, most likely built up by secretory components secreted into the matrix of the parasitophorous vacuole. In the absence of compound (Figure 1E,F), tachyzoites proliferated rapidly, forming large parasitophorous vacuoles containing numerous individual daughter tachyzoites ready to undergo egress and to infect neighboring host cells.

### 3.2. DAC-MS Overview

**Figure 2:**
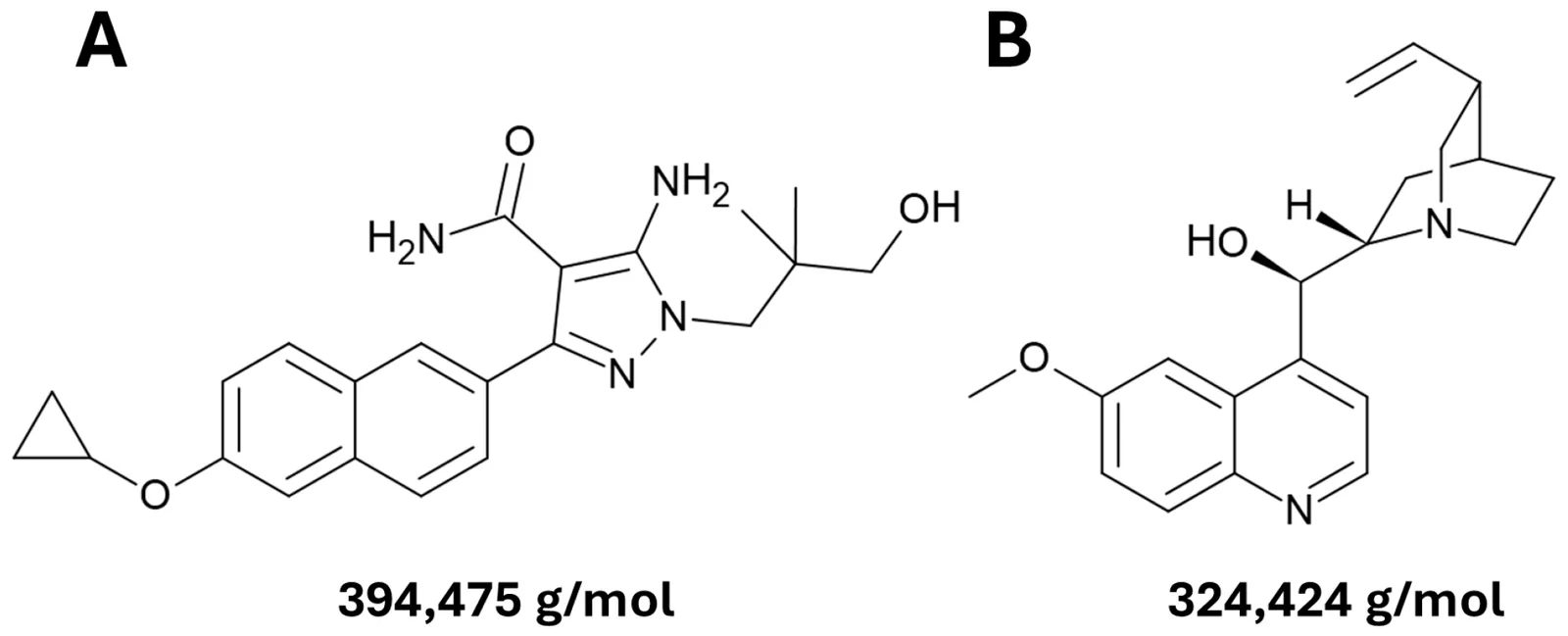
Chemical structures of BKI-1708 (**A**) and quinine (**B**) and their respective molecular weights.

Differential affinity chromatography (DAC) was performed using BKI-1708 as the compound of interest and quinine as the differential compound, given its structural similarity to BKI-1708. The two compound structures are shown in Figure 2, and the workflow applied in this study is schematically summarized in Supplementary Figure S1.

Mass spectrometry analysis of the proteomes obtained after DAC of cell-free extracts from *T. gondii* ME49 tachyzoites and HFF yielded a total of 14,897 unique peptides matching 453 *T. gondii* proteins and 9873 unique peptides matching 831 host cell proteins. The complete datasets for HFF (infected and non-infected) are provided in Table S1, while the dataset for *T. gondii* is provided in Table S2. Visualization of the protein intensity distributions (PID) by box plots shows that they exhibit some degree of variation between the different samples (see Supplementary Figure S2), and hierarchical clustering shows that *T. gondii*-infected HFF samples segregated significantly from non-infected samples (Supplementary Figure S3).

### 3.3. T. gondii Proteins Binding to BKI-1708 and/or Quinine Identified by DAC-MS

**Figure 3:**
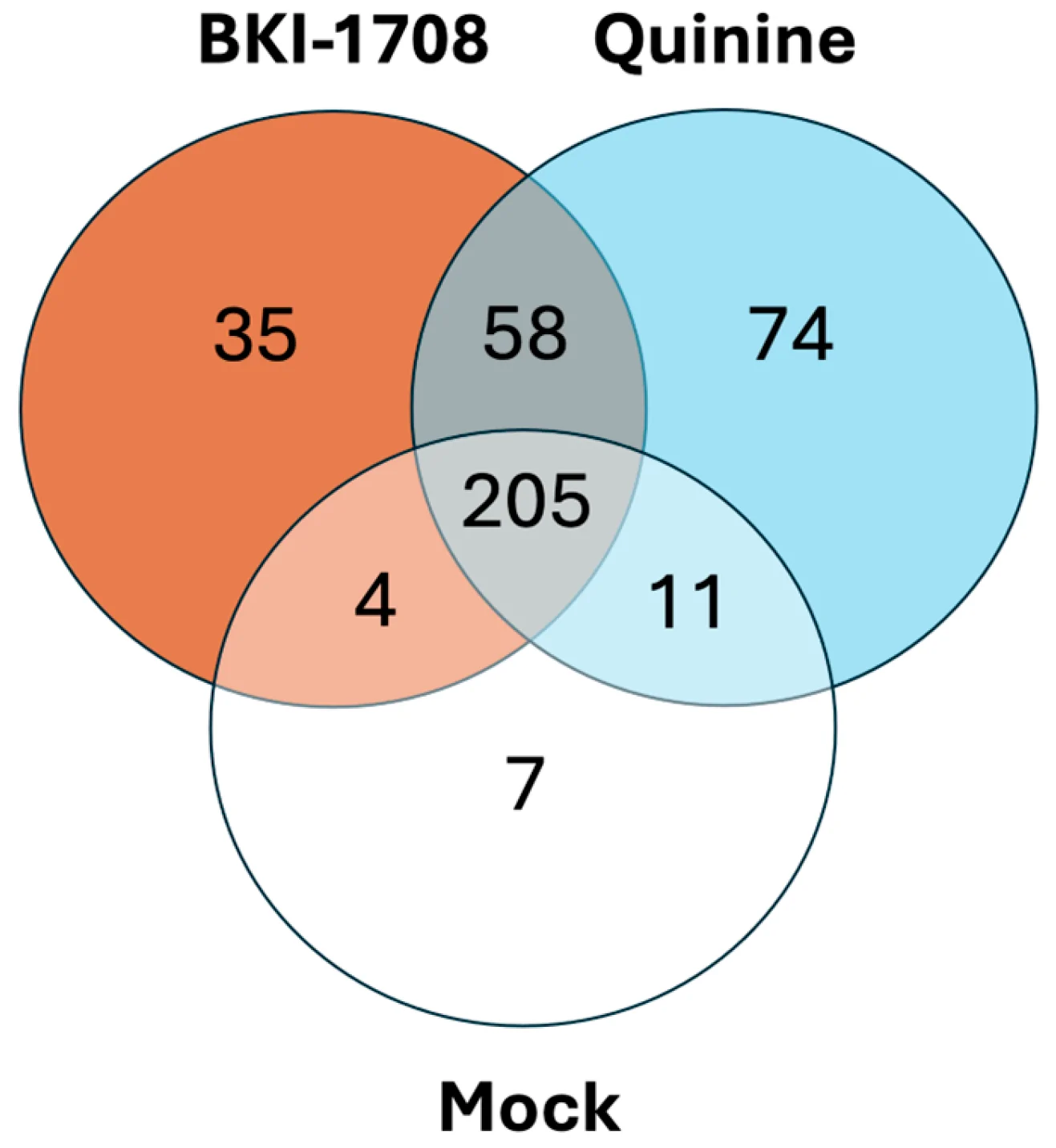
Venn diagram detailing the number of proteins binding to BKI-1708 (brown), quinine (blue) and mock columns (white), identified by DAC in cell-free extracts of *T. gondii* ME49 tachyzoites. The eluates from BKI-1708 and quinine columns were compared by MS shotgun analysis as described in Section 2. The number of proteins within the subsets is explained in detail in the text.

A total of 453 *T. gondii* proteins were identified by DAC (Figure 3; Table S2). 205 proteins were identified in all three eluates from BKI-1708, quinine, and mock columns. 35 proteins were specifically eluted from the BKI-1708 column, and they are listed in (Table 1). A total of 132 proteins were detected in the quinine-binding fraction, of which 58 were also identified in the BKI-1708 column fraction (Table 2), and 74 proteins were found to bind specifically to quinine.

The proteins binding specifically to BKI-1708, shown in Table 1, are assigned to a variety of cellular functions and pathways linked to cell division, cytoskeleton, vesicular trafficking, secretory organelles, detoxification, and transcriptional and translational regulation (Table 1). Among those, mitochondrial peroxiredoxin 3 (TgPrx3), which is involved in managing oxidative stress and modulates the immune response during infection [33], exhibited the by far highest relative abundance, followed by the putative protein transport TgSEC31. Other components of the BKI-1708-specific fraction are proteins with transport functions and nucleic acid-binding activities, and secretory proteins involved in host–parasite interactions, such as dense granule proteins (GRA29, GRA62), rhoptry kinase family protein ROP25, and the perforin-like protein TgPLP1. Among others, the metacaspase TgMCA2, a member of the caspase-like cysteine protease family present in plants, fungi, and protozoa, and the AP2 domain transcription factor AP2VIII-2, a central regulator of transcription, were also among the specific BKI-1708 binders. The two previously described canonical BKI-targets, namely CDPK1 and MAPKL1, were not found within the BKI-1708 DAC dataset.

The 58 proteins retained by both BKI-1708 and quinine (20 of which are listed in Table 2) are involved in RNA metabolism, including splicing, translation, and ribosome biogenesis. This includes multiple RNA-binding proteins (RRM- and LSM domain-containing proteins), pre-mRNA processing factors (splicing factor 3b, TgPRPF19), nucleosome assembly protein (TgNAP), ribosome biogenesis regulators (TgRRS1, TgNOL1/NOP2), ribosomal protein TgL1, and rRNA pseudouridine synthase, and several components of the translation initiation machinery (TgeIF3 subunits E, 3, 7, 10; IF-2; TgeIF3 subunit 6–interacting protein), elongation-related factors (TgEF-1 GEF domain protein) and tRNA-associated proteins (tRNA import protein, glutamate-tRNA ligase). In addition, this fraction also contains proteins linked to energy metabolism and mitochondria (ATP synthase subunits α, β, F1γ; TgSDHB), cytoskeletal/structural elements (Tgcentrin 3, TgIMC33), vesicular trafficking (clathrin light chain and adaptor complex proteins), signaling/regulation (Tg14-3-3, TgCK2 kinase, TgRCC1, TgPPM3C), and secretory organelles (TgGRA23, TgGRA30, TgAMA1). The protein with the highest abundance is a hypothetical protein (TGME49_201860), whose expression levels were downregulated in *T. gondii* baryzoites [24].

### 3.4. DAC-MS Revealed Distinct BKI-1708 Affino-Proteomes in T. gondii-Infected and Non-Infected HFF

**Figure 4:**
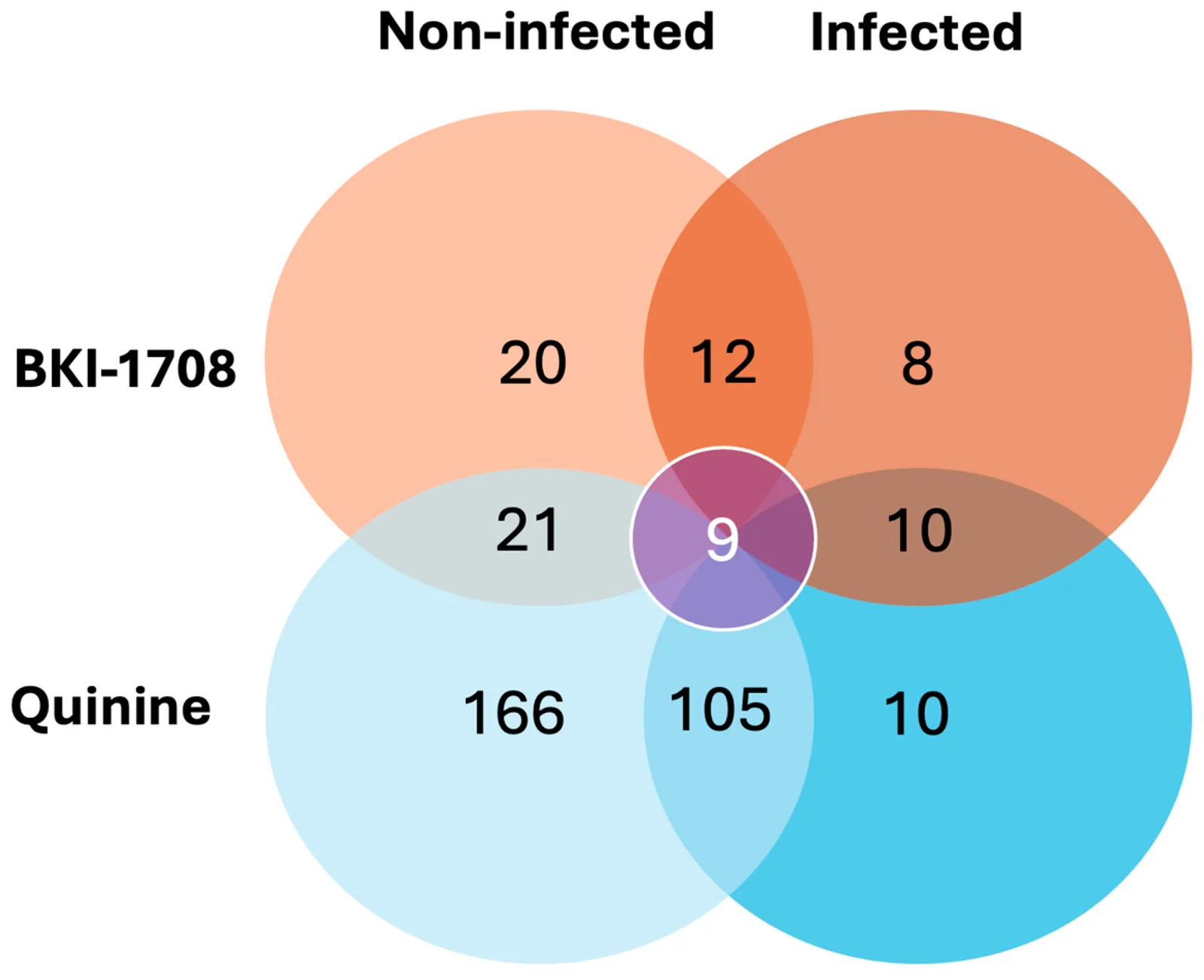
Venn diagram detailing the number of HFF proteins identified by DAC in cell-free extracts of not-infected and infected cells. Eluates from BKI-1708 and quinine columns were compared by MS shotgun analysis as described in Section 2. The number of proteins within the subsets is explained in detail in the text. Importantly, 9 proteins bind to BKI-1708 and quinine in both non-infected and *T. gondii*-infected HFF.

Amongst a total of 831 human host cell proteins identified by DAC, 434 proteins were eluted from the mock column. Subsets of the residual 397 specific compound-binding proteins identified in infected and non-infected cells are shown in Figure 4. BKI-1708 interacted with distinct protein subsets depending on the infection status of HFF: in non-infected cells, 20 proteins were specifically engaged, whereas eight proteins were exclusive to infected HFF. A set of 12 proteins was shared between both conditions, representing a core of infection-independent binders. Overlap with quinine controls was minimal, with only nine proteins common to all conditions.

#### 3.4.1. Host Cell Proteins Identified by DAC-MS in Non-Infected HFF

First, DAC-MS was performed using extracts from non-infected HFF. The largest subset of proteins, namely 166, was identified exclusively in eluates from quinine columns. The full list of proteins can be accessed in Table S1. Overall, quinine-specific binding proteins included 21 ribosomal proteins, three Rab proteins, and 25 transport-related proteins involved in protein synthesis and trafficking. Additionally, 34 proteins identified were associated with the cytoskeleton, including actin, tubulin, catenins, and myosins, while 43 proteins are involved in phosphorylation, dephosphorylation, signal relay, GTP-binding, and receptor activity. Fifteen proteins of the total subset were linked to energy metabolism. Fractions containing proteins binding to both BKI-1708 and to quinine contained a clearly diminished subset of 21 binding proteins (Table 3), and the fraction of proteins binding specifically to BKI-1708 comprised 20 proteins (Table 4).

The three most abundant HFF proteins binding to both BKI-1708 and quinine (Table 3) were F-actin-capping protein subunit beta (CAPZB), PDZ, and LIM domain protein 7 (PDLIM7), and the nuclear transport protein–protein SEC13 homolog (SEC13).

Out of the total of twenty proteins found interacting exclusively with BKI-1708 in non-infected cells (Table 4), the most abundant protein found was translation machinery-associated protein 7 (TMA7), followed by Stathmin (STMN1). In addition, several BKI-1708 specific binding proteins are involved in cytoskeletal regulation and intracellular trafficking, including (i) PDZ and LIM domain protein 1 (PDLIM1); (ii) isoform 2 of Dihydropyrimidinase-related protein 2 (DPYSL2); (iii) isoform 10 of protein transport protein Sec31A (Sec31A); (iv) SLAIN motif-containing protein 2 (SLAIN2); and (v) microtubule-associated proteins 4 (MAP4) and 2 (MAP2). Furthermore, annexins A11 and A7 (ANXA11, ANXA7) were also identified as BKI-1708-specific binders. Identified BKI-1708 binders directly involved in RNA-related processes include RNA-binding CCHC-type zinc finger nucleic acid-binding protein (CNBP) and eukaryotic translation initiation factor 4 gamma 2 (eIF4G2). Furthermore, two proteins with DNA-binding functions were identified in the BKI-1708 affino-proteome dataset: WD repeat-containing protein 5 (WDR5) and Protein SET (SET). The third most abundant identified protein is Secreted Ly-6/uPAR-related protein 1 (SLURP1). In addition, CREB-regulated transcription coactivator 3 (CRTC3) was identified as a protein that binds specifically to BKI-1708. Lastly, Catalase (CAT), which regulates the degradation of hydrogen peroxide, was found to bind specifically to BKI-1708 (Table 4).

#### 3.4.2. Proteins Identified by BKI-1708 DAC-MS in *T. gondii*-Infected HFF

In *T. gondii*-infected host cells, the number of proteins binding to BKI-1708 was markedly reduced when compared to non-infected conditions. Specifically, eight proteins were found to interact exclusively with BKI-1708, eleven bound only to quinine, and ten were identified in both BKI-1708 and quinine column eluates (Figure 4, Table 5 and Table 6).

Amongst the eight host proteins uniquely associated with BKI-1708 in infected cells, the most abundant was the secreted protein-binding extracellular glycoprotein lacritin (LACRT) (Table 5), followed by isoform 2 of polyglutamine-binding protein 1 (PQBP1-2). Beyond these, the proteins binding exclusively to BKI-1708 in infected cells were associated with nucleic acid and protein metabolism, with only a single protein (mitochondrial L-lactate dehydrogenase B chain (LDHB) linked to energy metabolism. Moreover, two nuclear proteins were detected: isoform 2 of nuclear pore complex protein Nup153 (NUP153) and isoform 2 of pescadillo homolog (PES1). Other pulled-down proteins with RNA-binding activities include (i) isoform 2 of Myb-binding protein 1A (MYBBP1A) and (ii) isoform 2 of proliferation marker protein Ki-67 (MKI67). Lastly, isoform 2 of suprabasin (SBSN) was identified.

#### 3.4.3. Host Cell Proteins Binding Exclusively to BKI-1708 in Both *T. gondii*-Infected and Non-Infected HFF

Twelve proteins, mostly RNA-binding proteins, were found to bind specifically to BKI-1708 in both infected and non-infected cells (Table 7). Zinc finger protein 706 and Isoform 2 of splicing factor 1 (SF1) were the most abundant BKI-binder proteins detected in both eluates.

#### 3.4.4. Host Cell Proteins Binding to Both BKI-1708 and Quinine in *T. gondii*-Infected and Non-Infected HFF

In addition, a subset of nine proteins was identified in all column eluates, interacting with BKI-1708 and quinine in both infected and non-infected cells (Table 8), including RNA-binding proteins, proteins associated with cytoskeletal organization, intracellular transport, and functions in redox homeostasis.

### 3.5. Functional Assignments of T. gondii Tachyzoite and HFF Proteins Specifically Binding to BKI-1708

The putative functions of proteins that specifically bind to BKI-1708 in *T. gondii* ME49 tachyzoites, as well as in infected and non-infected host cell extracts, and their respective numbers, are shown in Table 9. Their functions were identified based on information available in ToxoDB (www.toxodb.org) and Uniprot (www.uniprot.org) databases.

In *T. gondii*, the five most abundant proteins found in BKI-1708 eluates include two proteins with catalytic activity related to oxidation-reduction reactions (Peroxiredoxin PRX3 and Oxidoreductase, 2OG-Fe(II) oxygenase family protein), one hypothetical protein (TGME49_209420), and two proteins involved in protein transport (protein transport SEC31 and SEC13). Secreted proteins involved in host cell interaction and parasitism were also engaged by BKI-1708: two dense granule proteins (GRA29 and GRA62), rhoptry kinase family protein ROP25, and Perforin-like protein PLP1. Overall, *T. gondii* proteins identified interacting only with BKI-1708 cover functions related to cytoskeleton dynamics, nucleic acid binding, oxidative stress response, and intracellular transport.

## 4. Discussion

Exposure of *T. gondii* tachyzoites to BKI-1708 induces the formation of MNCs, also known as baryzoites, which represent a drug-induced stage that contains newly formed zoites blocked in the final stages of cytokinesis, remaining viable for extended periods of time [24]. In order to identify proteins that bind specifically to BKI-1708, DAC-MS was performed employing BKI-1708 and the related compound quinine, on cell-free extracts of *T. gondii* tachyzoites, *T. gondii*-infected HFF, or non-infected HFF.

Although DAC-MS has been used successfully as an approach for the identification of drug targets, it is important to consider that there are some intrinsic limitations [25]. DAC-MS favors the detection of more abundant proteins, while proteins expressed at low levels might not be picked up. In addition, the direct binding of a soluble protein to a drug does not inherently demonstrate functional inhibition by the drug, or that binding also occurs under native conditions or in vivo. In addition, proteins recovered from DAC-MS experiments may represent: (i) direct binders present as intact, functional proteins; (ii) proteins rendered accessible through cell lysis, including those from disrupted host or parasite compartments or degraded material; and (iii) nonspecific, high-abundance or “sticky” contaminants. Nevertheless, several potential molecular targets for BKI-1708 were identified in *T. gondii* and in infected and uninfected HFF host cells, suggesting that this compound could act through perturbation of multiple cellular processes rather than a single dominant target.

### 4.1. BKI-1708-Binding Proteins in T. gondii Are Associated with Multiple Molecular Functions

205 *T. gondii* proteins exhibited unspecific binding to BKI-1708, quinine, and the mock column. The 58 proteins retained by both BKI-1708 and quinine are largely involved in RNA metabolism, translation, and ribosome biogenesis. Overall, this suggests that both BKI-1708 and quinine associate with a broad set of housekeeping processes, particularly post-transcriptional regulation and translational control, with additional links to metabolism and intracellular trafficking, and that these interactions are likely based on the 5-amido-caboxamide group common to both drugs. The lack of noticeable anti-parasitic activity of quinine against *T. gondii* [34] could indicate that recognition of this core motif alone is insufficient for parasite inhibition, and that additional structural features might be required.

In total, 35 proteins were exclusively identified in the BKI-1708 affino-proteome. They likely reflect additional, side-chain-mediated interactions that confer potency and/or parasite selectivity. BKI-1708 was bound to proteins associated with secretory organelles, vesicular trafficking, redox regulation, and parasite-specific transcriptional regulation, consistent with a more specialized and potentially invasion-related binding profile. The most abundant protein, TgPRX3, is known to have antioxidant activity [33] and is involved in modulating immunopathology during *T. gondii* infection [35]. Secretory components such as ROP and GRA proteins are known to facilitate host cell entry and modify host cell functions, and the perforin-like protein TgPLP1 is necessary for efficient parasite egress [36]. The metacaspase TgMCA2, also binding specifically to BKI-1708, is implicated in apoptosis-like cell death [37]. Previous studies have shown that TgMCA2, in conjunction with TgMCA1, is critical for parasite replication and pathogenicity, and the lack of these proteins affected IMC1 maturation, impaired endodyogeny, increased bradyzoite differentiation, and reduced virulence in mice [38]. The AP2 domain transcription factor AP2VIII-2 was also identified as a specific BKI-1708 binder. AP2 factors are central regulators of transcription in *T. gondii* and govern mechanisms that repress bradyzoite differentiation during the tachyzoite stage [39]. Recent work has shown that AP2VIII-2, in conjunction with other factors, formed an essential chromatin-remodeling complex that maintains transcriptional fidelity, enabling stage-specific gene expression [40].

While CDPK1 and MAPKL1 were not found within the DAC datasets, two additional specific BKI-1708 binders had been found recently to be mutated in BKI-1748-resistant *T. gondii* [17]. Specifically, mutations were detected in TgPLU-1 in one out of six resistant strains and in Tubulin-tyrosine ligase family protein in three out of six strains [17]. Although the functional characterization of PLU-1 in *T. gondii* remains limited, PLU-1 family proteins in mammalian systems have been associated with transcriptional repression, suggesting a potential regulatory role [41,42]. Tubulin-tyrosine ligase family protein was predicted to be a tubulin polyglutamylase involved in microtubule polyglutamylation [43], and was increasingly expressed during cell division and localized to the daughter IMC [44].

Specific BKI-1708 binders also included five proteins that had been found earlier to be expressed at lower abundancies in *T. gondii* tachyzoites treated with 2.5 μM BKI-1708 [24]: (i) two hypothetical proteins (TGME49_313270, TGME49_205320); (ii) cold-shock DNA-binding domain-containing protein; (iii) putative eukaryotic translation initiation factor 3 subunit G that specifically targets and initiates translation of a subset of mRNAs involved in cell proliferation; and (iv) a zinc finger (CCCH-type) motif-containing protein. Notably, the latter was detected in untreated tachyzoites but was absent in BKI-1708-treated parasites [24]. CCCH-type zinc fingers are well known to bind RNA rather than DNA and play key roles in RNA processing, stability, and translation regulation, thereby contributing to post-transcriptional control of gene expression [45]. The downregulation or loss of these proteins in baryzoites suggests that they may represent direct binding targets of the compound, or alternatively, components of protein complexes disrupted by its presence. Such changes could be either a direct consequence of compound binding—through mechanisms such as protein degradation or transcriptional repression—or an indirect result of a shutdown of associated pathways. Another finding was that the most highly abundant protein binding to both quinine and BKI-1708, the hypothetical protein TGME49_201860, had also been found to be downregulated in *T. gondii* baryzoites [24].

### 4.2. Host Cell Drug Binding Proteomes in Non-Infected HFF and T. gondii-Infected HFF Exhibit Distinct Differences

The results obtained in HFF showed that both the compounds (BKI-1708 and/or quinine) and the HFF infection status had an influence on the protein composition of respective pull-down fractions. Nevertheless, a small set of nine proteins was identified that bind to both BKI-1708 and quinine, regardless of the infection status. This set included RNA-binding proteins, as well as proteins involved in cytoskeletal organization, intracellular transport, and redox homeostasis. The overlap between the two affinity proteomes of BKI-1708 and quinine reflects a shared affinity for the 5-amido-caboxamide core structure again. In addition, not only quinine, but also BKI-1708, displayed compound-specific binding: twenty host proteins were uniquely detected in pull-downs from extracts of non-infected HFF, and eight additional host proteins interacted exclusively with BKI-1708 in extracts of *T. gondii*-infected cells.

Out of the total of twenty proteins found to interact exclusively with BKI-1708 in non-infected HFF, the two most abundant proteins were translation machinery-associated protein 7, with predicted functions in protein synthesis in humans, followed by the microtubule-destabilizing protein isoform 2 of stathmin [46,47]. Additionally, several other proteins involved in cytoskeletal regulation and intracellular trafficking were found to bind specifically to BKI-1708, including (i) PDZ and LIM domain protein 1 and isoform 2 of dihydropyrimidinase related protein 2 [48,49] involved in signaling and subsequent remodeling of the cytoskeleton; the transport protein Sec31A, which promotes vesicle formation from the endoplasmic reticulum [50]; and SLAIN-motif containing protein 2, MAP2, and MAP4, all involved in regulating microtubule growth, assembly, and/or stability [51,52,53]. The annexins ANXA11 and ANXA7 display reported functions associated with membrane dynamics, the cytoskeleton, and RNA binding [54,55], as do the CCHC-type zinc finger nucleic acid-binding protein [56] and the eukaryotic translation initiation factor 4 gamma 2 [57]. The DNA-binding proteins WD repeat-containing protein 5 and SET are involved in histone modification [58], transcription, apoptosis, nucleosome assembly, and histone chaperoning [59,60]. The third most abundant protein secreted, Ly-6/uPAR-related protein 1, exhibits antitumor activity [61]. CRTC3 acts as a coactivator that enhances CREB transcriptional activity in response to cellular signals such as stress or metabolic changes [62,63].

Catalase, also exclusively bound by BKI-1708, catalyzes the degradation of hydrogen peroxide, thus protecting cells from its toxic effects [64]. Interestingly, *T. gondii*, PRX3, with similar antioxidant functions, was also found to bind specifically to BKI-1708, with the highest rAbu of the set. In addition, while the second-most abundant BKI-1708 and quinine binding protein in uninfected HFF was a SEC13 homolog, the second-most abundant specific BKI-1708 binder in *T. gondii* was the transporter TgSec3. Overall, this indicates the presence of shared molecular targets in parasites and host cells, and that BKI-1708-binding proteins in *T. gondii* and non-infected HFF seem to be associated with largely similar pathways. This mirrors earlier findings on comparative DAC-MS using a closely related compound, BKI-1748, identifying respective binding proteins in *N. caninum* tachyzoite and zebrafish extracts [27]. In both organisms, a majority of BKI-1748 binding proteins were involved in RNA binding and modification, in particular, splicing, and eluates from both organisms contained proteins involved in DNA binding or modification and key steps of intermediate metabolism. However, while the BKI-1748 DAC-MS of *Neospora* resulted in the identification of NcCDPK1 [27], one of the validated BKI-targets, as a drug-binding protein; neither TgCDPK1 nor TgMAPKL1 was identified as a drug binder in this study. This could be because these proteins might interact with the drug only transiently, or binding conditions were suboptimal, leading to unstable interactions, or due to the lack of sensitivity of the method if these proteins are present only at low concentrations. Other explanations include immobilization chemistry, altered active-site accessibility, lysis conditions, or EDTA effects on calcium-dependent interactions. In any case, BKI-1748 interacted with not only specific targets in apicomplexans, such as CDPK1, but also with targets in other eukaryotes, which are involved in common, essential pathways [27], and the same appears true for BKI-1708.

*T. gondii*-infected HFF had markedly fewer readouts overall compared to non-infected HFF, which may reflect limitations related to sample availability due to cell lysis or altered host cell protein composition upon *T. gondii* infection. In summary, eight proteins were found to interact exclusively with BKI-1708 in *T. gondii*-infected HFF, eleven proteins were specifically bound to quinine, and ten were identified in both BKI-1708 and quinine column eluates. In contrast to non-infected HFF, the specific BKI-1708 binders in *T. gondii*-infected cells were mostly DNA and RNA-interacting factors. The most abundant was the secreted protein-binding extracellular glycoprotein lacritin, typically associated with lacrimal and salivary tissues, which plays a role in immune regulation and tear secretion [65]. The second most abundant protein was isoform 2 of polyglutamine-binding protein 1, which is involved in pre-mRNA splicing, transcriptional regulation, innate immunity, and neuronal development [66,67]. The only protein linked to energy metabolism is the mitochondrial L-lactate dehydrogenase B chain, which is associated with catalyzing the stereospecific interconversion of pyruvate and lactate coupled to the NADH/NAD^+^ redox system [68]. Beyond these, the proteins binding exclusively to BKI-1708 in infected cells were associated with nucleic acid and protein metabolism, such as isoform 2 of nuclear pore complex protein Nup153 and isoform 2 of pescadillo homolog, which is essential for rRNA processing and 60S ribosome biogenesis [69,70]. Other pulled-down proteins with RNA-binding activities include isoform 2 of Myb-binding protein 1A [71] and isoform 2 of proliferation marker protein Ki-67 [72]. Lastly, isoform 2 of suprabasin was identified, although its functional role remains unclear.

Overall, proteins involved in functions associated with the cytoskeleton, intracellular signaling, and transport are absent in the BKI-1708 affino-proteome of *T. gondii*-infected HFF, possibly reflecting that infection modulates host signaling and chromatin-related pathways. It is known that *T. gondii* profoundly reprograms the host proteome and metabolism, with metabolic rewiring for parasite benefit, immune signaling modulation, and structural/cellular adjustments to support intracellular survival [73,74]. Host cytoskeletal regulators and actin dynamics are essential for *T. gondii* invasion and intracellular growth [75], making the host trafficking machinery and actin/microtubule-associated proteins critical for parasite establishment and survival. These infection-induced changes can profoundly alter the accessibility of host proteins. RNA-binding proteins, however, remain engaged regardless of infection status.

A core set of twelve proteins was bound specifically to BKI-1708 in both *T. gondii*-infected and non-infected HFF. Proteins captured by BKI-1708 in both conditions were mostly involved in RNA binding or were not annotated and thus designated as hypothetical proteins. Few are functionally involved in stress-related pathways. One of the most abundant proteins identified was isoform 2 of splicing factor 1, which is reported to bind to RNA and function in the early stages of pre-mRNA splicing [76,77]. In a previous study investigating the molecular targets of BKI-1748 using DAC-MS in *C. parvum* and host cells, isoform 2 of SF1 was also found to bind to both BKI-1748 and quinine in the human colon tumor cell line HCT-8 [28]. In the present study, isoform 2 of SF1 was identified in HFF as a specific binder of BKI-1708 but not of quinine, suggesting a potentially unique role for SF1 in the mechanism of action of BKI-1708. The compound may have a chemical structure or binding mode that allows it to selectively engage SF1, in contrast to BKI-1748. Alternatively, differences between host cell lines—such as SF1 expression levels, post-translational modifications, interacting partners, or subcellular localization—could influence the ability of SF1 to interact with BKI-1708 or quinine.

### 4.3. Caveats and Limitations of DAC-MS Proteomics

It is important to point out that proteins recovered from DAC-MS experiments may represent direct or indirect binders, and although quinine was used as a control drug with a similar structure but without noticeable effect, unspecific interactions cannot be excluded completely. In addition, even direct binding of a protein to a drug does not inherently demonstrate functional inhibition by the drug, or that binding also occurs under native conditions or in vivo. For instance, the presence of EDTA in a protease inhibitor cocktail included in the lysis buffer could influence calcium-dependent interactions, as for CDPK1, but also other proteins. Evidently, the fact that HFF do not exhibit any obvious impairment in viability upon exposure to BKI-1708 suggests that they have, in addition to the above possibilities, evolved mechanisms to circumvent potential adverse effects, at least at the concentrations used in this study. Lastly, kinase targets may be underrepresented depending on immobilization chemistry and active-site accessibility [25].

Despite these intrinsic methodological limitations, results presented herein suggest that in *T. gondii* BKI-1708 has a broader impact beyond canonical kinase targets, including (i) engagement of secretory organelles (ROP25, TgGRA29, TgGRA62, and micronemal TgPLP1); (ii) binding to components of vesicular trafficking machinery (TgSEC13/23/31); (iii) interaction with transcriptional/epigenetic control elements (TgAP2VIII-2, CHD1/SWI2/SNF2, PLU-1); (iv) redox regulation (TgPRX3, TgTLAP4); and (v) cytoskeleton-associated proteins (TgIMC25).

**Figure 5:**
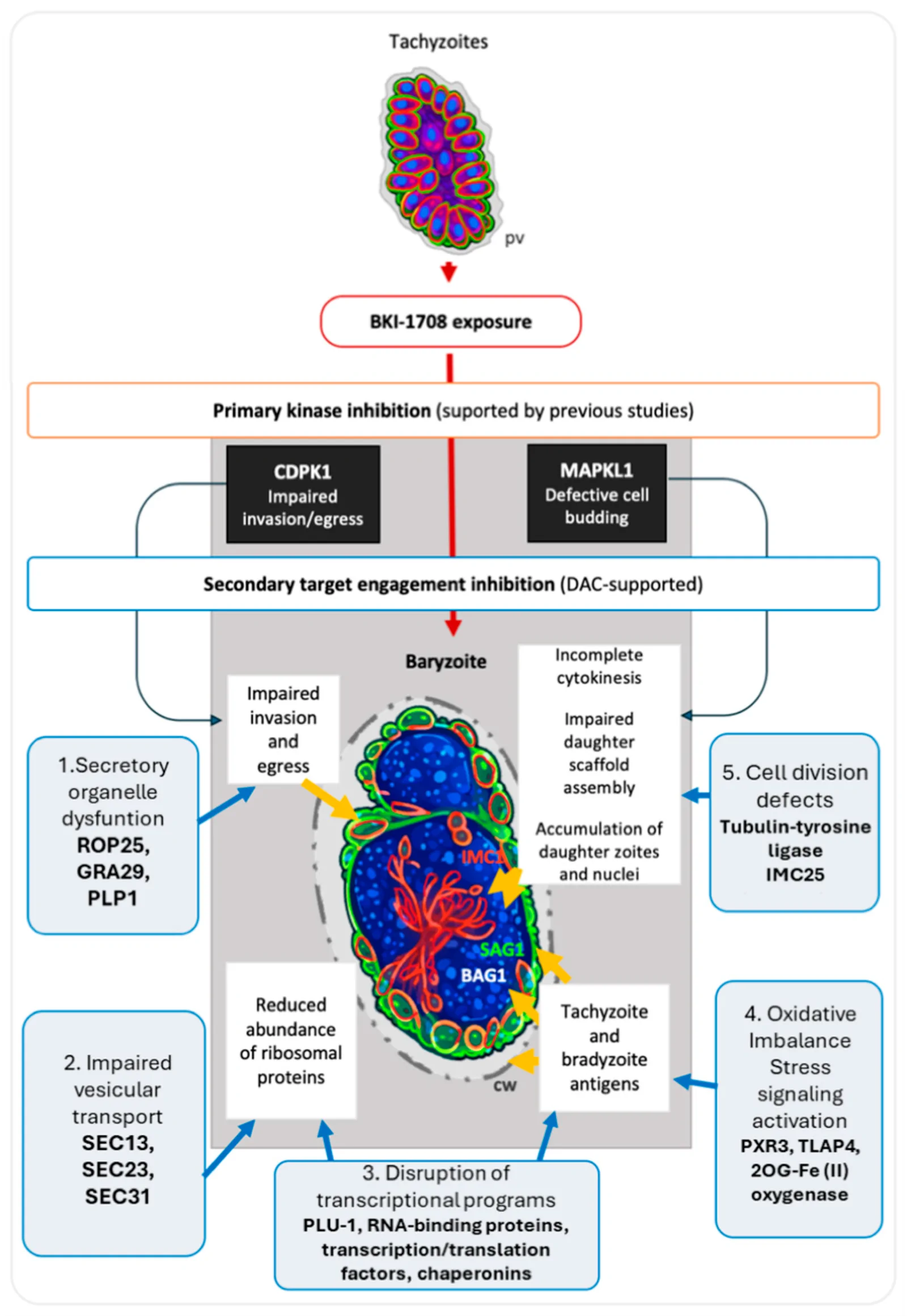
Schematic representation of proposed BKI-1708-mediated multi-layered disruption of *Toxoplasma gondii* tachyzoites leading to baryzoite formation. As shown in [24], aberrant baryzoites display mixed stage identity, co-expressing the tachyzoite marker surface antigen SAG1 and the bradyzoite antigen BAG1, with altered IMC organization and peripheral deposition of cyst wall-like material (cw). Proposed molecular targets of BKI-1708 are indicated in boxes with a gray background, and the corresponding potential cellular effects are indicated in boxes with a white background.

This binding profile suggests that BKI-1708 could perturb protein trafficking, organelle biogenesis, stress homeostasis, and gene expression networks simultaneously. Collectively, these multilayered disruptions provide a mechanistic basis for defective cytokinesis, organelle segregation failure, and the emergence of multinucleated baryzoites under drug pressure. Consistent with this, as mentioned, BKI-1708-treated tachyzoites failed to complete normal division and egress. It is conceivable that this phenotype results from multi-target interactions, including RNA binding and modulation of cell cycle-related proteins, disrupting coordinated processes required for parasite replication and egress. Figure 5 schematically summarizes plausible drug-disrupted pathways based on BKI-engaged proteins, possibly leading to the observed baryzoite phenotype under BKI-1708 treatment. The findings that BKI-1708 could potentially interact with multiple protein targets align with the emerging view that antiprotozoal drugs often operate via multiple mechanisms rather than through a single, well-defined target [25].

## 5. Conclusions

In *T. gondii* and in non-infected HFF, DAC-MS has identified potential molecular targets of BKI-1708, such as proteins involved in RNA regulation, cytoskeleton dynamics, and intracellular transport. However, these putative targets remain to be validated. Infection of HFF with *T. gondii* altered the host protein binding profile, with no engagement of cytoskeletal proteins, while RNA-binding proteins remained consistently engaged regardless of infection status. In non-infected HFF and *T. gondii* tachyzoites, cytoskeletal and RNA-binding proteins represent core molecular targets of its activity. In *T. gondii* tachyzoite extracts, BKI-1708 engagement also extends to additional proteins involved in egress and invasion. Together, these findings provide a mechanistic explanation for the impaired cytokinesis phenotype observed in baryzoites induced by BKI treatment, and point towards a multifactorial mode of action of BKI-1708.

## Figures and Tables

**Table 1:** List of the 35 *T. gondii* ME49 proteins specifically binding to BKI-1708. The relative abundances (rAbu) based on iBAQ sum up to a total of 1,000,000 for each sample. The proteins are listed according to their decreasing rAbu values.

Toxo DB ORF	Annotation	rAbu
TGME49_230410	peroxiredoxin PRX3	604.9
TGME49_311400	protein transport protein SEC31, putative	368.6
TGME49_290920	oxidoreductase, 2OG-Fe(II) oxygenase family protein	251.6
TGME49_209420	hypothetical protein	248.3
TGME49_201700	protein transport protein SEC13	232.4
TGME49_224720	SPOC domain-containing protein	216.6
TGME49_205180	RNA recognition motif-containing protein	190.9
TGME49_313270	hypothetical protein	174.3
TGME49_320600	cold-shock DNA-binding domain-containing protein	172.5
TGME49_232370	CW-type Zinc Finger protein	160.7
TGME49_273960	chaperonin GroS protein	151.5
TGME49_231440	LsmAD domain-containing protein	137.2
TGME49_294670	eukaryotic translation initiation factor 3 subunit G, putative	127.0
TGME49_263530	chaperonin, putative	91.1
TGME49_265250	RNA recognition motif-containing protein	84.3
TGME49_202780	rhoptry kinase family protein ROP25	74.6
TGME49_250830	26S proteasome regulatory subunit RPN12, putative	73.9
TGME49_201760	thioredoxin-like associated protein TLAP4	61.1
TGME49_218240	inner membrane complex protein IMC25	54.4
TGME49_278975	metacaspase MCA2	51.5
TGME49_205320	hypothetical protein	48.2
TGME49_291680	protein transport protein SEC23, putative	44.4
TGME49_309200	zinc finger (CCCH type) motif-containing protein	41.6
TGME49_204160	GYF domain-containing protein	39.1
TGME49_269690	dense granule protein GRA29	36.0
TGME49_250115	hypothetical protein	21.1
TGME49_204130	perforin-like protein PLP1	20.0
TGME49_232280	hypothetical protein	18.6
TGME49_215360	dense granule protein GRA62	17.9
TGME49_258240	chromodomain helicase DNA binding protein CHD1/SWI2/SNF2	10.7
TGME49_298610	GYF domain-containing protein	9.6
TGME49_233120	AP2 domain transcription factor AP2VIII-2	8.6
TGME49_253750	PLU-1 family protein	7.3
TGME49_254940	MIF4G domain-containing protein	7.3
TGME49_244500	Tubulin-tyrosine ligase family protein	2.8

**Table 2:** List of the 20 most abundant *T. gondii* ME49 proteins binding to both BKI-1708 and quinine columns. The relative abundances (rAbu) based on iBAQ sum up to a total of 1,000,000 for each sample. The proteins are listed according to their decreasing rAbu values in BKI-1708 eluates.

Toxo DB ORF	Annotation	rAbu BKI-1708	rAbu Quinine
TGME49_201860	hypothetical protein	1185.1	600.9
TGME49_205558	NAC domain-containing protein	827.0	53.9
TGME49_244110	nucleosome assembly protein (nap) protein	300.3	34.8
TGME49_314830	pre-mRNA splicing factor subunit, putative	202.3	160.9
TGME49_232000	dense granule protein GRA30	190.2	43.6
TGME49_248250	translation initiation factor IF-2, putative	175.8	376.6
TGME49_294970	hypothetical protein	156.6	38.8
TGME49_265530	RNA recognition motif-containing protein	130.9	8.9
TGME49_257380	inhibitor of cysteine protease 1	122.5	181.9
TGME49_263090	14-3-3 protein	115.4	50.4
TGME49_500284	hypothetical protein, conserved	88.8	13.9
TGME49_291330	RNA recognition motif-containing protein	88.1	105.8
TGME49_260670	centrin 3	86.9	61.6
TGME49_213030	clathrin light chain, putative	84.2	437.1
TGME49_213940	CHCH domain-containing protein	78.1	35.8
TGME49_280550	clathrin adaptor complex small chain subfamily protein	74.7	77.3
TGME49_300280	LSM domain-containing protein	64.5	48.0
TGME49_269180	MIF4G domain-containing protein	60.4	4.5
TGME49_213050	hypothetical protein	60.2	41.2
TGME49_313640	hypothetical protein	51.6	166.6

**Table 3:** List of host cell proteins binding to BKI-1708 and quinine in non-infected cells. The relative abundances (rAbu) based on iBAQ sum up to a total of 1,000,000 for each sample. The proteins are listed according to their decreasing rAbu values in BKI-1708 column eluates of non-infected cells.

Protein ID	Annotation	rAbu BKI-1708	rAbu Quinine
P47756	F-actin-capping protein subunit beta	164.5	663.0
P55735	Protein SEC13 homolog	161.5	351.3
Q9NR12	PDZ and LIM domain protein 7	156.5	210.0
P52907	F-actin-capping protein subunit alpha-1	147.5	741.8
Q07021	Complement component 1 Q subcomponent-binding protein, mitochondrial	135.2	151.0
Q8WX93-3	Isoform 3 of Palladin	109.7	11.8
P51608-2	Isoform B of Methyl-CpG-binding protein 2	92.1	45.6
P09493-8	Isoform 8 of Tropomyosin alpha-1 chain	71.6	463.0
P51659	Peroxisomal multifunctional enzyme type 2	60.5	18.0
Q96GY0	Zinc finger C2HC domain-containing protein 1A	60.3	31.4
Q9C0C2	182 kDa tankyrase-1-binding protein	50.5	160.2
Q8ND56-2	Isoform 2 of Protein LSM14 homolog A	49.4	64.1
Q16531	DNA damage-binding protein 1	35.4	18.0
Q13428-2	Isoform 2 of Treacle protein	31.0	143.0
Q9HAU0-2	Isoform 2 of Pleckstrin homology domain-containing family A member 5	28.7	63.5
Q14974	Importin subunit beta-1	18.6	449.9
P08123	Collagen alpha-2(I) chain	15.2	20.9
P50281	Matrix metalloproteinase-14	12.1	55.5
P49821-2	Isoform 2 of NADH dehydrogenase [ubiquinone] flavoprotein 1, mitochondrial	11.6	25.4
O60664-3	Isoform 3 of Perilipin-3	9.3	192.6
O14974-5	Isoform 5 of Protein phosphatase 1 regulatory subunit 12A	9.2	185.0

**Table 4:** List of host cell proteins specifically binding to BKI-1708 columns in non-infected cells. The relative abundances (rAbu) based on iBAQ sum up to a total of 1,000,000 for each sample. The proteins are listed according to their decreasing rAbu values.

Protein ID	Annotation	rAbu
Q9Y2S6	Translation machinery-associated protein 7	5041.5
P16949-2	Isoform 2 of Stathmin	1608.4
P55000	Secreted Ly-6/uPAR-related protein 1	324.7
P50995-2	Isoform 2 of Annexin A11	286.3
P82909	Alpha-ketoglutarate dehydrogenase component 4	195.2
Q96FJ2	Dynein light chain 2, cytoplasmic	160.2
P01036	Cystatin-S	157.5
P62633-2	Isoform 2 of CCHC-type zinc finger nucleic acid-binding protein	128.7
Q01105	Protein SET	77.4
O00151	PDZ and LIM domain protein 1	66.2
P61964	WD repeat-containing protein 5	62.6
P11137-2	Isoform 2 of Microtubule-associated protein 2	61.7
Q6UUV7-3	Isoform 3 of CREB-regulated transcription coactivator 3	57.6
Q16555-2	Isoform 2 of Dihydropyrimidinase-related protein 2	49.7
Q9P270	SLAIN motif-containing protein 2	47.5
O94979-10	Isoform 10 of protein transport protein Sec31A	40.5
P20073-2	Isoform 2 of Annexin A7	30.0
P78344	Eukaryotic translation initiation factor 4 gamma 2	27.7
P04040	Catalase	26.7
P27816-5	Isoform 5 of Microtubule-associated protein 4	24.6

**Table 5:** List of proteins specifically binding to BKI-1708 in *T. gondii*-infected HFF. The relative abundances (rAbu) based on iBAQ sum up to a total of 1,000,000 for each sample. The proteins are listed according to their decreasing rAbu values in BKI-1708 eluates of infected cells.

Protein ID	Annotation	rAbu
Q9GZZ8	Extracellular glycoprotein lacritin	244.7
O60828-2	Isoform 2 of Polyglutamine-binding protein 1	76.5
P07195	L-lactate dehydrogenase B chain	43.1
Q6UWP8-2	Isoform 2 of Suprabasin	26.1
P49790-2	Isoform 2 of Nuclear pore complex protein Nup153	23.7
O00541-2	Isoform 2 of Pescadillo homolog	23.6
Q9BQG0-2	Isoform 2 of Myb-binding protein 1A	22.6
P46013-2	Isoform Short of Proliferation marker protein Ki-67	8.7

**Table 6:** List of proteins of *T. gondii*-infected HFF binding to both BKI-1708 and quinine. The relative abundances (rAbu) based on iBAQ sum up to a total of 1,000,000 for each sample. The proteins are listed according to their decreasing rAbu values in BKI-1708 eluates of infected cells.

Protein ID	Annotation	BKI-1708 rAbu	Quinine rAbu
P11387	DNA topoisomerase 1	97.2	27.2
Q9NX24	H/ACA ribonucleoprotein complex subunit 2	85.5	98.7
O75400-2	Isoform 2 of Pre-mRNA-processing factor 40 homolog A	45.0	7.0
Q9NXV6	CDKN2A-interacting protein	30.4	9.7
P78316	Nucleolar protein 14	21.4	5.7
P31943	Heterogeneous nuclear ribonucleoprotein H	17.1	139.2
Q8NDZ4	Divergent protein kinase domain 2A	16.7	2.8
P00750-2	Isoform 2 of Tissue-type plasminogen activator	12.3	17.4
O94776-2	Isoform 2 of Metastasis-associated protein MTA2	11.1	15.3
Q8WUM4-2	Isoform 2 of Programmed cell death 6-interacting protein	10.3	2.4

**Table 7:** List of proteins binding exclusively to BKI-1708 in both *T. gondii*-infected and non-infected HFF. The relative abundances (rAbu) based on iBAQ sum up to a total of 1,000,000 for each sample. The proteins are listed according to their decreasing rAbu values in BKI-1708 eluates of non-infected cells.

Protein ID	Annotation	rAbu Non-Infected	rAbu Infected
Q9Y5V0	Zinc finger protein 706	645.0	55.9
Q15637-2	Isoform 2 of Splicing factor 1	456.8	584.5
P78406	mRNA export factor RAE1	257.1	136.8
Q6E0U4-16	Isoform 16 of Dermokine	228.9	127.8
O43684-2	Isoform 2 of Mitotic checkpoint protein BUB3	213.9	44.1
Q13492-2	Isoform 2 of Phosphatidylinositol-binding clathrin assembly protein	194.8	66.7
O75223-3	Isoform 3 of Gamma-glutamylcyclotransferase	192.0	149.3
Q9NPA8-2	Isoform 2 of Transcription and mRNA export factor ENY2	171.9	101.7
Q8WWM7-2	Isoform 2 of Ataxin-2-like protein	157.0	167.9
P0CG12	Decreased expression of renal and prostate cancer protein	75.6	30.3
P22234-2	Isoform 2 of Bifunctional phosphoribosylaminoimidazole carboxylase/phosphoribosylaminoimidazole succinocarboxamide synthetase	15.3	51.0
Q96AE4-2	Isoform 2 of Far upstream element-binding protein 1	13.7	154.3

**Table 8:** List of proteins binding to BKI-1708 and quinine in both non-infected and *T. gondii*-infected HFF. The relative abundances (rAbu) based on iBAQ sum up to a total of 1,000,000 for each sample. The proteins are listed according to their decreasing rAbu values in BKI-1708 eluates of infected cells.

Protein ID	Annotation	Non-Infected HFF		Infected HFF	
		BKI-1708 rAbu	Quinine rAbu	BKI-1708 rAbu	Quinine rAbu
P35637-2	Isoform Short of RNA-binding protein FUS	571.8	257.7	391.0	69.1
Q14011	Cold-inducible RNA-binding protein	506.4	299.3	203.4	63.9
Q9H0D6-2	Isoform 2 of 5′-3′ exoribonuclease 2	16.8	41.5	65.1	18.2
P63167	Dynein light chain 1, cytoplasmic	791.7	1136.8	41.2	42.5
Q9UHB6-4	Isoform 4 of LIM domain and actin-binding protein 1	8.7	584.4	20.7	2.4
Q06830	Peroxiredoxin-1	458.2	246.8	19.5	53.7
Q9UN86-2	Isoform B of Ras GTPase-activating protein-binding protein 2	41.5	81.4	15.9	16.1
P12956-2	Isoform 2 of X-ray repair cross-complementing protein 6	19.3	61.7	11.3	56.0
Q8WWI1-3	Isoform 3 of LIM domain only protein 7	20.3	137.5	6.2	1.7

**Table 9:** Summary of putative functions and respective numbers of host cell and *T. gondii* proteins binding specifically to BKI-1708. The molecular processes assigned to the different functions are listed in Table S3.

Function	Uninfected HFF Proteins	Infected HFF Proteins	Infected and Uninfected Host Cell Proteins	*T. gondii* ME49 Proteins
DNA binding and modification	2	3	0	5
RNA binding and modification	4	2	5	6
Protein binding and modification	3	1	1	5
Cytoskeleton and intracellular transport	6	0	1	4
Intracellular signaling	2	0	0	5
Energy and intermediary metabolism	0	1	2	2
Hypothetical or ambiguous	3	1	3	6
Total	20	8	12	35

## Data Availability

The original contributions presented in this study are included in the article/Supplementary Materials. Further inquiries can be directed to the corresponding authors.

## Supplementary Materials

The following supporting information can be downloaded at: https://www.mdpi.com/article/10.3390/microorganisms14081608/s1, Table S1: HFF binding proteins; Table S2: *Toxoplasma* binding proteins; Table S3: Functional categorization of binding proteins; Figure S1: Summary of the workflow applied in this study, including (1) protein extraction and processing, (2) differential affinity chromatography using BKI-1708 and a structurally related but inactive compound (quinine), and (3) LC-MS and database mining; Figure S2: Protein intensity distributions (PIDs) represented by boxplots of the log2-transformed IBAQ (intensity based absolute quantification). Cell-free extracts of non-infected HFF (H—human) and infected HFF (T—Toxoplasma) were prepared and subjected to differential affinity chromatography on mock (HM1, HM2, TM1, TM2), quinine (HQ, TQ), or BKI-1708 (HB, TB) columns. Eluates were analyzed by mass spectrometry and proteomics as described in Section 2; Figure S3: Hierarchical clustering of the log2-transformed IBAQ intensities. Cell-free extracts of non-infected HFF (H—human) and infected HFF (T—Toxoplasma) were subjected to differential affinity chromatography on mock (HM1, HM2, TM1, TM2), quinine (HQ, TQ), or BKI-1708 (HB, TB) columns. Eluates were analyzed by mass spectrometry and proteomics as described in Section 2.
