# Supplementary material for: Affino-Proteomic Analysis of Bumped Kinase Inhibitor BKI-1708 in Toxoplasma gondii and Human Fibroblast Host Cells
Source: Microorganisms. 2026 Jul 23;14(8):1608. doi: 10.3390/microorganisms14081608 (PMC13515763; doi:10.3390/microorganisms14081608)
Supplement: Supplementary file 1 [file microorganisms-14-01608-s001.zip › Supplementary Figure S1.pdf]

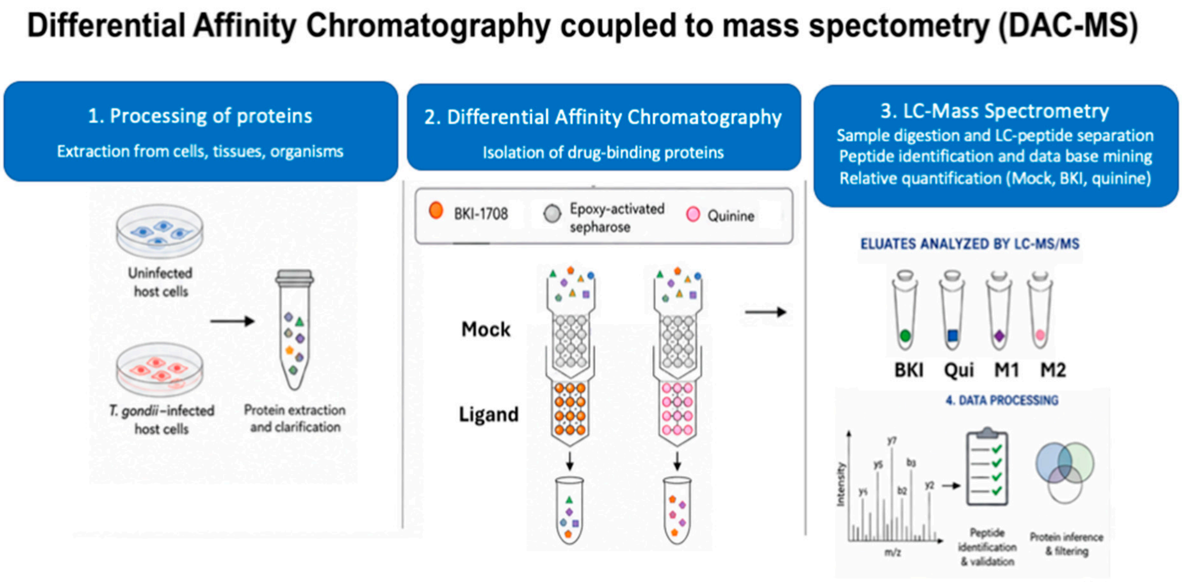

Supplementary Figure S1. Summary of the workflow applied in this study, including (1) protein extraction and processing, (2) differential affinity chromatography using BKI-1708 and a structurally related but inactive compound (quinine), and (3) LC-MS and database mining.
