# Supplementary material for: Affino-Proteomic Analysis of Bumped Kinase Inhibitor BKI-1708 in Toxoplasma gondii and Human Fibroblast Host Cells
Source: Microorganisms. 2026 Jul 23;14(8):1608. doi: 10.3390/microorganisms14081608 (PMC13515763; doi:10.3390/microorganisms14081608)
Supplement: Supplementary file 1 [file microorganisms-14-01608-s001.zip › Supplementary Figure S2.pdf]

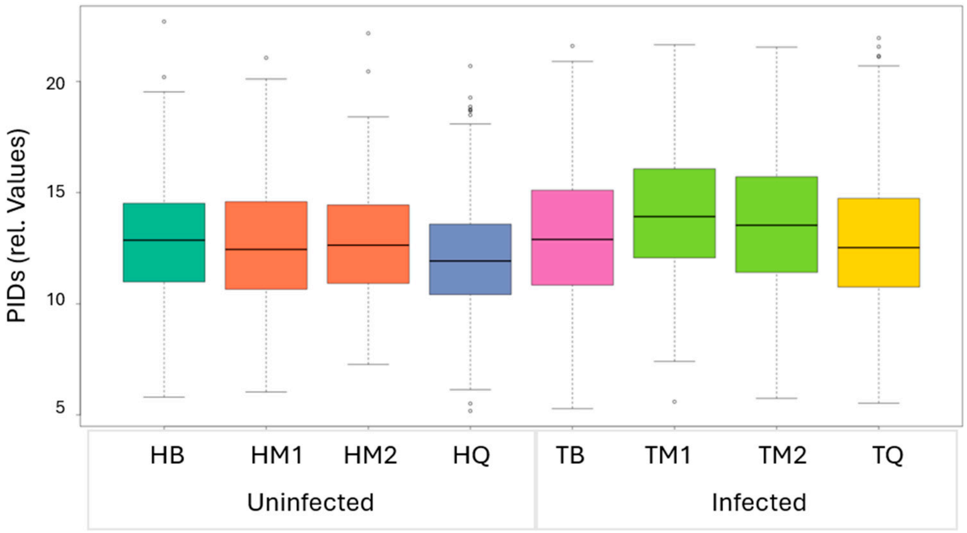

Supplementary Figure S2. Protein intensity distributions (PIDs) represented by boxplots of the log2-transformed IBAQ (in-tensity based absolute quantification). Cell-free extracts of non-infected HFF (H - human) and infected HFF (T – *Toxoplasma*) were prepared and subjected to differential affinity chromatography on mock (HM1, HM2, TM1, TM2), quinine (HQ, TQ), or BKI-1708 (HB, TB) columns. Eluates were analyzed by mass spectrometry and proteomics as described in Section 2.
